# The Impact of Sarcopenia, Myosteatosis, and Visceral Adiposity on Renal Transplantation Outcomes

**DOI:** 10.3390/medicina61091608

**Published:** 2025-09-05

**Authors:** Esin Olcucuoglu, Utku Eren Ozkaya, Muhammed Emin Polat, Mehmet Yılmaz, Sedat Tastemur, Rıza Sarper Okten, Erkan Olcucuoglu

**Affiliations:** 1 Ministry of Health, Ankara Bilkent City Hospital, Department of Radiology, Ankara 06800, Turkey; sarperokten@yahoo.com; 2 Ministry of Health, Izmir Cigli Research and Training Hospital, Izmir 35030, Turkey; utkuerenozkaya@gmail.com; 3 Ministry of Health, Ankara Bilkent City Hospital, Department of Urology, Ankara 06100, Turkey; emnplt25@gmail.com (M.E.P.); sedattastemur@yahoo.com (S.T.); erkanolcucuoglu@gmail.com (E.O.); 4 MediClin Kraichgau-Klinik, Urology, 74906 Bad Rappenau, Germany; yilmazmehmet88@hotmail.com

**Keywords:** renal transplantation, outcomes, myosteatosis, sarcopenia, visceral adiposity

## Abstract

*Background and Objectives*: The impact of sarcopenia and myosteatosis on renal transplantation (RT) outcomes has yet to be explained, certainly due to differences in assessment methods. The role of visceral adiposity is also not clearly defined. This retrospective study aimed to evaluate pretransplant body composition—including sarcopenia, myosteatosis, and visceral adiposity ratio (VSR)—using computed tomography (CT) and analyze their relationship with short- and long-term graft outcomes. *Materials and Methods*: A total of 94 patients who underwent RT between 2019 and 2023 and had pretransplant non-contrast abdominal CT scans were included. Skeletal muscle area (SMA) was assessed at the L3 vertebral level, including multiple muscle groups. Sarcopenia was defined by a low skeletal muscle index (SMI), while myosteatosis was defined by high intramuscular adipose tissue content (IMAC). Visceral adiposity was evaluated by the visceral-to-subcutaneous adipose tissue ratio (VSR). These parameters were compared with post-transplant outcomes. *Results*: The mean age was 42.69 ± 12.47 years, with 54.3% male patients. High IMAC was significantly associated with early graft failure (*p* = 0.026), delayed graft function (*p* = 0.005), death-censored graft failure (*p* = 0.036), and overall graft failure (*p* = 0.047). One-year mortality was also higher in the high IMAC group (14.8% vs. 0.0%, *p* = 0.012). SMI and VSR were not significantly associated with outcomes. Myosteatosis emerged as a significant risk factor in univariate analysis but was not independently predictive in multivariate analysis. Among the established risk factors identified in the study, recipient age was found to be a significant predictor for overall graft failure, donation type (cadaveric vs. living) for death-censored graft failure, and cold ischemia time for delayed graft function (OR: 1.068, 95% CI: 1.001–1.141, *p* = 0.049; OR: 147.7, 95% CI: 2.1—10,427.0, *p* = 0.021; OR: 1.003, 95% CI: 1.001–1.006, *p* = 0.023). *Conclusions*: Myosteatosis correlates with worse graft outcomes and higher mortality, but its independent prognostic value requires further investigation.

## 1. Introduction

The kidney is the most frequently transplanted solid organ, with renal transplantation (RT) recognized as the most effective treatment for improving survival and quality of life in patients with end-stage renal failure (ESRF) [1]. Death with a functioning graft is the leading cause of kidney allograft loss [2]. While factors such as recipient age, duration of dialysis, comorbidities (including diabetes mellitus (DM) and cardiovascular diseases (CVD)), and donor characteristics are well-established risk factors, new potential contributors to mortality and morbidity in RT need to be further explored and identified.

**Figure 1:**
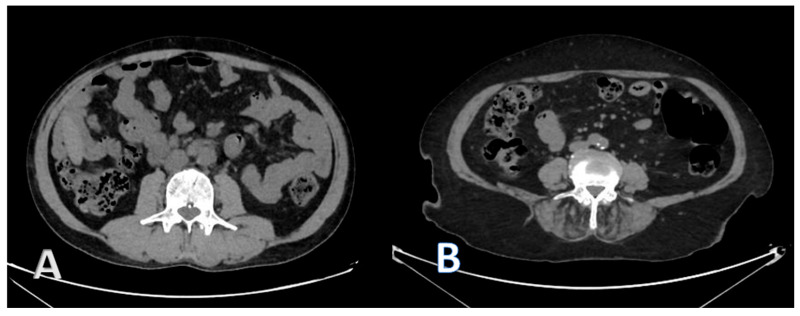
CT image at the L3 level of a recipient with normal muscle mass and quality (**A**) and a recipient with sarcopenia and myosteatosis (**B**).

Sarcopenia is now recognized as a condition characterized by the progressive loss of muscle mass and strength, which leads to impaired quality of life, increased disability, frailty, and mortality [3]. Beyond the natural aging process, many factors, such as a lack of exercise, chronic inflammation during dialysis, and vitamin D deficiency, play an important role in the development of sarcopenia in patients with ESRF [4]. In addition to sarcopenia, patients with chronic kidney disease may also experience a qualitative deterioration in muscle structure, such as myosteatosis and fatty infiltration (Figure 1) [5]. However, the reported associations between sarcopenia or myosteatosis and RT outcomes have shown variability across studies, primarily due to differences in analysis techniques and threshold definitions used to quantify muscle mass and quality [6,7,8,9,10]. In our study, we aimed to reduce this heterogeneity by using computed tomography (CT)-based assessments at the L3 level and applying standardized, sex-specific cut-off values for skeletal muscle index (SMI), intramuscular adipose tissue content (IMAC), and visceral-to-subcutaneous adipose tissue ratio (VSR), as proposed by Hamaguchi et al. [11].

CT is widely utilized for body composition analysis, allowing for the assessment of skeletal muscle and adipose tissue as well as the differentiation of visceral and subcutaneous fat [12]. Visceral adiposity, represented by the VSR, is calculated by dividing the visceral adipose tissue (VAT) area by the subcutaneous adipose tissue area. Emerging research suggests that a high VSR is a useful predictor of poor outcomes in liver transplantation [11]. However, the impact of high VSR on RT outcomes remains largely unknown.

High visceral fat levels can contribute to myosteatosis by causing a decrease in muscle mass and an increase in fat content in muscles, and can also facilitate the development of sarcopenia by negatively affecting muscle metabolism through the secretion of inflammatory cytokines [13]. In this study, we demonstrated the feasibility of jointly examining SMI, IMAC, and VSR using CT, which enabled a more comprehensive assessment of different dimensions of body composition. While previous studies have typically focused on one or two of these parameters, combining all three in this study may facilitate the development of more effective risk assessment methods for clinical applications and provide a new perspective on understanding complications following kidney transplantation.

Therefore, in our study, we retrospectively measure pretransplant muscularity (sarcopenia and myosteatosis) and VSR using CT in patients undergoing RT. We then analyze the impact of body composition measurements on short- and long-term graft outcomes following RT. To our knowledge, this is the only RT study in which three risk factors, accurately measurable by CT, are evaluated simultaneously.

## 2. Materials and Methods

### 2.1. Data Collection

Approval was obtained from the Ankara Bilkent City Hospital Ethics Committee (approval no: E2-23-5324). All adult kidney transplant recipients (KTRs) who had undergone RT at our institution between March 2019 and June 2023 and had a non-contrast abdominal CT scan available within 3 months before surgery were included in the study. Moreover, 28 patients with insufficient follow-up data (less than 6 months), 1 patient who underwent combined liver-kidney transplantation, 9 patients under the age of 18, 21 patients lacking a preoperative CT scan, and 4 patients with conditions preventing accurate evaluation (e.g., subcutaneous edema) were excluded from the study. Consequently, of the 157 KTRs, 94 were included in the analysis. Clinical data, including sex, recipient age, Body mass index (BMI), comorbidities, duration of dialysis, donor type (deceased or living), and duration of cold ischemia, were collected from medical records. BMI was calculated as weight (kg) divided by height (m^2^). The primary exposures assessed were sarcopenia (low SMI), myosteatosis (high IMAC), and visceral adiposity (high VSR). To evaluate the impact of body composition parameters on clinical outcomes, we specifically examined their associations with both short-term outcomes (early graft failure, delayed graft function, and acute rejection) and long-term outcomes (graft function decline, death-censored graft failure, overall graft failure, and mortality). Logistic regression analyses were performed to assess whether sarcopenia (low SMI), myosteatosis (high IMAC), and visceral adiposity (high VSR) were predictors of these outcomes after adjusting for established risk factors. Additional clinical variables such as recipient age, duration of dialysis, donor type, and duration of cold ischemia were included in regression models as covariates for adjustment based on established literature.

### 2.2. Outcomes

Short-term graft outcomes were analyzed under early graft failure, delayed graft function, and acute rejection. Cases diagnosed by biopsy or clinically treated as acute rejection, despite not being eligible for biopsy, were recorded. Early graft failure was defined as returning to routine dialysis within 1 mo following RT [14], while delayed graft function was recorded as requiring transient dialysis within 1 week post-transplantation [15]. Long-term graft outcomes included graft function decline, death with a functioning graft, death-censored graft failure, and total graft failure. Graft function decline was defined as a decrease in the estimated glomerular filtration rate (e-GFR) of more than 10 mL/min/1.73 m^2^ between the first year after transplantation and the last follow-up [16]. Patients who died during follow-up without graft dysfunction or the need for dialysis were classified as having died with a functioning graft. Death-censored graft failure was defined as returning to dialysis or requiring re-transplantation due to graft dysfunction without death during follow-up. Total graft failure included all cases of graft loss, including death with a functioning graft [17].

### 2.3. CT Image Acquisition and Analysis

**Figure 2:**
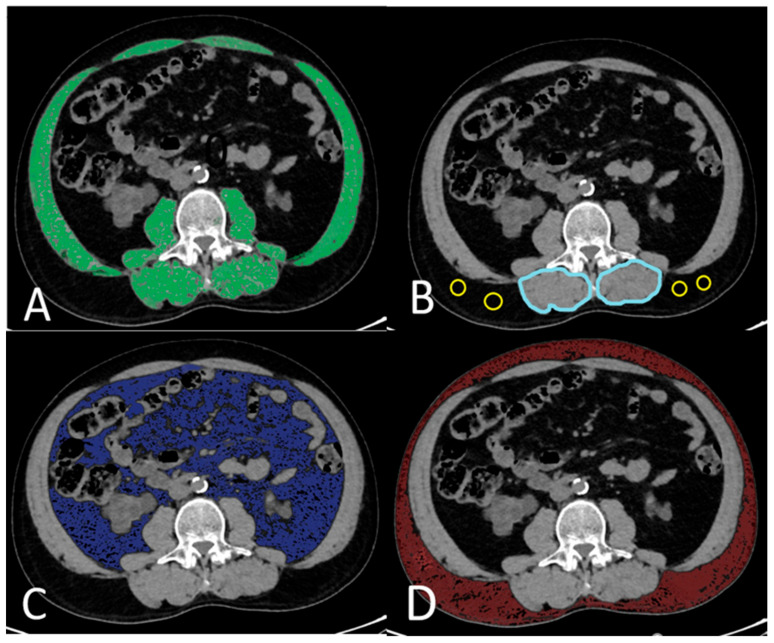
Cross-sectional computed tomographic images obtained at the L3 vertebral level. (**A**), SMA (green-painted area), including psoas, erector spinae, quadratus lumborum, transversus abdominis, external and internal obliques, and rectus abdominis, were identified and measured using −29 to 150 HU. (**B**), CT values of subfascial muscle tissue (2 large blue circles) and subcutaneous fat (4 small circles) in the multifidus muscle are examined to calculate IMAC. (**C**), VAT areas (blue-painted area) measured using −150 to −50 HU. (**D**), Subcutaneous adipose tissue areas (red-painted area) were measured using −190 to −30 HU. HU, Hounsfield units, SMA: Skeletal muscle area, VAT: Visceral adipose tissue.

Preoperative abdominal CT examinations were performed without contrast material, using a 128-slice CT scanner (General Electric, Milwaukee, WI, USA). Using topograms, two-way images of patients in the supine position, from the dome of the diaphragm to the symphysis pubis, were used. The acquisition protocol collimation was 2 mm, the rotation time was 0.6 s, pitch 1, the FOV was 40 cm, the tube voltage was 100–120 kV, and the tube current was 200–400 mA. The skeletal muscles (rectus abdominis, lateral and oblique abdominal muscles, psoas major, quadratus lumborum, erector spinae, and multifidus muscles) at the L3 vertebral level were evaluated on a single slice in the abdominal CT examinations of the patients using the software program on workstations (AW4.7_13.003_HELIOS_6.6.X, Milwaukee, WI, USA). The Hounsfield unit (HU) range of (−29)/(150) was chosen to determine the muscle structures (Figure 2A). The SMA was measured at the L3 vertebral level on a single section. The SMA was normalized to the square of the patient’s height, and an ‘SMI’ in cm^2^/m^2^ was obtained. The quality of skeletal muscle was examined using IMAC at the L3 vertebral level. The IMAC was calculated by dividing the density of the multifidus muscles (HU) by the density of subcutaneous fat (HU) (Figure 2B). A greater amount of adipose tissue in skeletal muscle indicated lower muscle quality and higher IMAC levels. Similarly to the determination of skeletal muscles, subcutaneous and VAT areas were quantified using ranges of −190 to −30 HU (Figure 2C) and −150 to −50 HU (Figure 2D), respectively. The VSR was calculated by dividing the VAT area by the subcutaneous adipose tissue area.

### 2.4. Body Composition Parameters

In our study, we used SMI, IMAC, and VSR cut-off values determined separately for men and women, as proposed by Hamaguchi et al. [11]. Defined cut-off values were used in our study because they are based on data obtained from CT scans of 657 healthy donors, calculated using standard deviation calculations, determined separately for men and women, and provide a broad and representative sample for determining the normal ranges of the parameters evaluated. The cut-off values for SMI were 40.31 cm^2^/m^2^ in men and 30.88 cm^2^/m^2^ in women. Similarly, the cut-off values for IMAC were −0.358 in men and −0.229 in women, while those for VSR were 1.325 in men and 0.710 in women [11]. The low SMI group was defined as sarcopenia, while the high IMAC group was characterized as myosteatosis. The group with high visceral adiposity was defined as a high VSR.

### 2.5. Statistical Analysis

The research data were evaluated using the SPSS 23.0 statistical package program. The conformity of the data to normal distribution was examined using visual (histogram) and analytical methods (Kolmogorov–Smirnov/Shapiro–Wilks tests). The descriptive analysis of the parametric data is presented using means and standard deviations, while nonparametric data are presented using median and interquartile range (IQR) (25–75) values. Chi-square tests were used for categorical variables as statistical methods, and Student’s *t* test or Mann–Whitney U test was used in comparisons between two independent groups. To evaluate the relationships between variables specified by the measurements, Pearson correlation was applied in cases of normal distribution, and Spearman correlation tests were applied in cases of nonnormal distribution. In comparisons of more than two independent groups, the significance was accepted as weak if the correlation coefficient was between 0 and 0.25, moderate if between 0.26 and 0.50, strong if between 0.51 and 0.75, and very strong if between 0.76 and 1.00. Logistic regression analysis was performed to determine the risk factors for short- and long-term graft outcomes, and the results are presented with odds ratios and 95% confidence intervals. In logistic regression, it is desired for the Nagelkerle R2 values, which show the degree of relationship between the dependent variable and the independent variables, to be high. This value is desired to be a minimum of 0.2. A *p*-value of less than 0.05 was accepted as statistically significant. In multivariate logistic regression analysis, in order to prevent overfitting, only variables that were significant at the *p* < 0.2 level in univariate analysis were included in the model. A backward stepwise method was applied to select independent predictors.

## 3. Results

The study was conducted with 94 patients, 54.3% (*n* = 51) of whom were male and 45.7% (*n* = 43) of whom were female. The average patient age was 42.69 ± 12.47 years. The median time from CT to transplantation was 13.5 d (IQR 25–75: 1–47), and the median follow-up time was 703.5 d (IQR 25–75: 440.75–1190.5).

Among the patients evaluated in the study, 70.2% (*n* = 66) were on hemodialysis before RT, 12.8% (*n* = 12) were on peritoneal dialysis before RT, and 17% (*n* = 16) were not routine dialysis patients and had undergone preemptive transplantation. Among the 78 patients receiving dialysis treatment, the total duration of dialysis ranged from 7.5 mo to 156 mo, with a median of 36 mo.

Of the transplants, 60.6% (*n* = 57) were performed from living donors and 39.4% (*n* = 37) were from cadaveric donors; 89.4% (*n* = 82) were performed in the right iliac fossa and 10.6% (*n* = 10) in the left iliac fossa. All arterial anastomoses were performed end-to-side to the external iliac artery. All venous anastomoses were performed with the external iliac vein. One renal artery anastomosis was performed in 75.6% of cases (*n* = 71), two in 22.3% (*n* = 21), and three in 2.1% (*n* = 2). When all transplants were evaluated, the duration of warm ischemia ranged from 120 to 170 s, with a median of 150 s. Cold ischemia time ranged between 110 and 40 min, with a median of 130 min.

Patient age was significantly higher in the high IMAC group (*p* = 0.003). BMI was lower in the low SMI group compared to the normal SMI group, but significantly higher in the high IMAC group than in the normal IMAC group (*p* = 0.001, *p* = 0.042). Median dialysis duration was 120 months in the high IMAC group and 15.5 months in the normal group, showing a significant correlation with IMAC (*p* = 0.003). Morbidity rates were significantly higher in the high IMAC group compared to the normal group (94.3% vs. 78%, *p* = 0.037). Donation type also differed significantly, with a higher proportion of living donors in the normal IMAC group (*p* = 0.023). The characteristics of patients classified according to SMI, IMAC, and VSR are shown in Table 1.

As shown in Table 2, the high IMAC group had significantly higher rates of early graft failure and delayed graft function (*p* = 0.026, *p* = 0.005). For long-term outcomes, death-censored and overall graft failure were more common in the high IMAC group (*p* = 0.036, *p* = 0.047). One-year mortality was significantly higher in the high IMAC group than in the normal group (14.8% vs. 0.0%, *p* = 0.012). No significant associations were observed between SMI or VSR groups and graft outcomes or mortality.

Regression analyses are summarized in Table 3. Univariate logistic regression revealed that recipient age (*p* = 0.049), dialysis duration (*p* = 0.004), cold ischemia time (*p* = 0.009), and myosteatosis (*p* = 0.042) were significant risk factors for early graft failure. For delayed graft function, significant factors were dialysis duration (*p* = 0.002), cold ischemia time (*p* = 0.001), and myosteatosis (*p* = 0.01). However, in multivariate analysis, myosteatosis did not remain an independent risk factor; cold ischemia time was the only significant factor (OR: 1.003, *p* = 0.023). For death-censored graft failure, univariate analysis identified donor age (*p* = 0.034), dialysis duration (*p* = 0.01), cadaveric donation (*p* = 0.007), cold ischemia time (*p* = 0.007), and myosteatosis (*p* = 0.034) as significant. Again, myosteatosis was not significant in multivariate analysis. Although the donation type appeared statistically significant (*p* = 0.021), its wide confidence interval (OR: 147.647, 95% CI: 2.091–10,427.038) limits its interpretation as an independent predictor. For overall graft failure, univariate analysis showed significance for recipient age (*p* = 0.046), dialysis duration (*p* = 0.010), cadaveric donation (*p* = 0.006), and cold ischemia time (*p* = 0.011). In multivariate analysis, recipient age remained the only independent risk factor (OR: 1.068, *p* = 0.049). Regarding 1-year mortality, univariate analysis identified dialysis duration (*p* = 0.016) and cold ischemia time (*p* = 0.025) as significant, but neither remained significant in multivariate analysis.

## 4. Discussion

Despite advancements in transplantation medicine, post-transplant graft failure and patient mortality remain key concerns [14]. Traditional risk factors, such as recipient age, comorbidities, and donor characteristics, are well established, but the influence of body composition on transplant success has been relatively understudied [14]. This study aimed to bridge this gap by evaluating the impact of sarcopenia, myosteatosis, and VSR on RT outcomes, offering a novel perspective on how pretransplant muscle and fat distribution may affect long-term graft function.

### 4.1. Visceral Adiposity

Adipose tissue, like skeletal muscle, is now recognized as a secretory organ and produces pro-inflammatory and anti-inflammatory cytokines and adipokines. Metabolic syndrome (MS) has been reported to be a clinical risk factor for post-transplant renal function [18]. VAT, a diagnostic marker of MS, is strongly associated with insulin resistance and CVD [19]. Studies have indicated that both VAT and VSR are predictors of CVD [20].

Excess visceral fat may impair renal function due to metabolic disturbances. Lee et al. demonstrated that VSR is significantly associated with delayed recovery of renal function, suggesting that it may serve as a predictor of renal function recovery in living kidney donors [21]. Emerging research has highlighted the endocrine, metabolic, and immunological functions of VAT, suggesting that visceral fat volume may be a more accurate measure than BMI in kidney disease [22]. Mitsui et al. emphasized that increased visceral fat ratio (VSR), assessed using 3D computed tomography, is strongly associated with post-transplant renal function decline in 58 living KTRs; this suggests that abdominal fat measurement may serve as an important prognostic tool for KTRs [23]. However, little data exist regarding the impact of VSR on post-transplant renal function. In the literature, there have been no studies addressing the impact of VSR on short- and long-term graft outcomes in recipients after RT, making our study a first in this regard. However, due to the limited number of patients in our study and the inclusion of both donor types (deceased/living), no significant relationship was found between VSR and graft outcomes.

### 4.2. Sarcopenia

Sarcopenia is highly prevalent among KTRs [4]. Numerous studies have linked sarcopenia to graft loss and mortality in RT when CT-based skeletal muscle assessments are used. Deliège et al. found that low muscle mass was associated with morbidity and mortality in older male RT recipients, using SMI at the L3 vertebra [6]. However, their study had limitations because it focused only on older male patients and used an SMI threshold that was not age-adjusted or derived from healthy individuals.

Druckmann et al. demonstrated that the psoas muscle cross-sectional area is an independent predictor of post-transplant mortality [7]. Additionally, Karakizlis et al. found that sarcopenia negatively impacts graft survival and long-term function [8].

Despite these findings, the effect of sarcopenia on RT outcomes remains uncertain, since some studies have shown conflicting results. A systematic review and meta-analysis by Zhang et al. reported no significant correlation between sarcopenia and rejection, infection, delayed graft function, or mortality [9]. Similarly, Tabourin et al. found no link between sarcopenia and complications within 1 year post-transplant, although low muscle density on CT was associated with late surgical complications [10].

In our study, no significant relationship was found between sarcopenia and RT complications or one-year mortality, supporting the existing uncertainty on this topic. The variability in methodologies, sample sizes, measurement techniques, and cut-off values used for diagnosing sarcopenia contributes to the conflicting results observed in the literature. Additionally, demographic differences among patients and the presence of comorbidities can alter the effects of sarcopenia on outcomes. For these reasons, reaching definitive and consistent conclusions regarding the impact of sarcopenia on RT remains challenging. Further studies are needed to standardize the methods and cut-off values used for diagnosing sarcopenia.

### 4.3. Myosteatosis

Myosteatosis, a marker of muscle quality, is commonly observed in patients with ESRF undergoing RT. Studies have indicated an association between myosteatosis and ESRF [24,25]. Risk factors for myosteatosis in RT recipients include age, prior RT, stroke history, and a BMI greater than 25 kg/m^2^ [26]. In our study, myosteatosis was significantly associated with older age, higher BMI, and prolonged dialysis duration (*p* = 0.003, *p* = 0.001, and *p* = 0.003, respectively).

Myosteatosis has been widely recognized as a predictor of mortality in various clinical settings, including cancer, liver transplantation, mechanical ventilation in intensive care, and even in healthy individuals without CVD who were followed for 11 years [27,28,29,30]. In the literature, a study conducted in non-dialysis chronic kidney disease patients has demonstrated that only myosteatosis, and not muscle mass, has a significant association with mortality in multivariable Cox regression models (Hazard Ratio 2.651, 95% CI 1.232–5.703, *p* = 0.013). The research indicates that myosteatosis is not sufficiently recognized in this patient group and may represent a significant risk factor [31]. Despite its established impact on other medical fields, its role in RT remains less explored.

A limited number of studies have investigated the effects of myosteatosis and a low SMI on post-transplant survival using a single unenhanced cross-sectional CT image at L3 [26,32]. Morel et al. identified myosteatosis as a significant risk factor for mortality within 4 years after RT and emphasized its prevalence among KTRs. Even after adjusting for other variables such as age, gender, and comorbidities, studies have shown that myosteatosis is an independent predictor of increased mortality after RT. Patients with myosteatosis had lower overall survival rates compared to those without myosteatosis, suggesting that myosteatosis may negatively impact long-term outcomes after RT. In this study, a more specific dataset was utilized, focusing primarily on the overall impact of myosteatosis on survival, without providing comments on complications [26].

According to Chen et al., myosteatosis (not sarcopenia) was an independent risk factor for mortality after RT, while neither myosteatosis nor sarcopenia was associated with graft loss. The presence of myosteatosis was associated with poorer functional outcomes and higher complication rates after RT. In contrast, Chen et al. employed a larger cohort and similarly demonstrated that myosteatosis is associated not only with mortality but also with early and late complications that may arise after RT [32].

In our study, similar to the other two studies, myosteatosis was found to be significantly associated with a higher 1-year mortality rate. However, regression analysis did not validate myosteatosis as an independent risk factor for these complications, indicating that further studies are needed to clarify its role in RT prognosis. Although multivariate logistic regression was employed to adjust for confounding variables, the relatively small sample size limited the inclusion of multiple covariates in each model. Also, some studies have suggested IMAC as an independent predictor, while others argue that it may simply reflect systemic metabolic or inflammatory changes such as obesity, insulin resistance, or chronic inflammation [33]. In our study, IMAC correlated strongly with dialysis duration and BMI, suggesting it may act more as a correlate of these risk factors rather than an independent marker. In light of this information, we should aim to reduce myosteatosis and improve transplant outcomes in patients with chronic kidney disease, particularly those with ESRF, through targeted interventions such as nutritional optimization, physical rehabilitation, and exercise-based strategies. However, further research is needed.

This study has several limitations. First, it was conducted retrospectively at a single institution, which may limit the generalizability of the findings. Second, the criteria and cut-off values used to define myosteatosis and sarcopenia varied, which could have influenced the results. Additionally, the relatively small sample size may have introduced bias, affecting the statistical power of the study.

## 5. Conclusions

Despite the limited sample size, the simultaneous assessment of sarcopenia, myosteatosis, and visceral adiposity in the same cohort using standardized cut-off values provides a new perspective that has not been extensively explored in previous studies. This study provides important insights into the impact of body composition parameters on RT outcomes. While sarcopenia and visceral adiposity were not significantly associated with graft function or survival, myosteatosis emerged as a notable risk factor for poorer outcomes, including higher 1-year mortality rates, early graft failure, and delayed graft function. However, its independent prognostic significance remains uncertain, emphasizing the need for further research with larger multicenter cohorts.

The findings suggest that myosteatosis may be an important factor to consider in the treatment of KTRs following RT, potentially guiding interventions aimed at improving muscle health and patient outcomes. Given the increasing evidence regarding the role of myosteatosis in transplantation success, its incorporation into pre-transplant assessments could enhance risk stratification and assist in personalized treatment planning. Ultimately, a multidisciplinary approach that integrates nutritional, metabolic, and imaging evaluations could optimize post-transplant care and improve long-term graft survival. Additionally, greater emphasis should be placed on care strategies that enhance overall health by preserving muscle mass in KTRs, such as balanced nutrition, regular exercise, and weight management. This not only aims to improve individual patient outcomes but also reduces the burden on healthcare systems.

Future prospective large cohort studies should aim to better understand the effects of myosteatosis by investigating diagnostic criteria, pathophysiological mechanisms, and potential intervention strategies in greater depth, thereby ensuring a more consistent and accurate assessment of myosteatosis in clinical practice.

## Figures and Tables

**Table 1:** Characteristics of patients classified according to SMI, IMAC, and VSR.

		SMI			IMAC			VSR		
	All (*n* = 94)	Low (*n* = 24)	Normal (*n* = 70)	*p*	High (*n* = 35)	Normal (*n* = 59)	*p*	High (*n* = 28)	Normal (*n* = 66)	*p*
Recipient age Mean (SD)	42.69 (±12.47)	41.29 (±12.08)	43.17 (±12.66)	0.527 *	47.6 (±10.80)	39.78 (±12.56)	0.003 *	46.25 (±10.03)	41.18 (±13.15)	0.071 *
Gender										
Female *n* (%)	43 (45.7%)	10 (58.3%)	33 (47.1%)	0.642 **	15 (42.9%)	28 (47.5%)	0.665 **	13 (46.4%)	30 (45.5%)	0.931 **
Male *n* (%)	51 (54.3%)	14 (41.7%)	37 (52.9%)		20 (57.1%)	31 (52.5%)		15 (53.6%)	36 (54.5%)	
BMI Median (IQR)	23.59 (20.56–26.81)	21.48 (19.32–23.25)	24.45 (21.96–27.88)	0.001 ***	24.24 (22.56–27.78)	22.65 (20.22–25.36)	0.042 ***	23.89 (21.81–27.29)	23.15 (20.31–26.56)	0.329 ***
Dialysis										
Pre-emptive *n* (%)	16 (17%)	3 (12.5%)	13 (18.6%)	0.931 ****	3 (8.6%)	13 (22%)	0.191 **	3 (10.7%)	13 (19.7%)	0.433 ****
Hemodialysis *n* (%)	66 (70.2%)	18 (75%)	48 (68.6%)		26 (74.3%)	40 (67.8%)		20 (71.4%)	46 (69.7%)	
Peritoneal Dialysis *n* (%)	12 (12.8%)	3 (12.5%)	9 (12.9%)		6 (10.2%)	6 (17.1%)		5 (17.9%)	7 (10.6%)	
Dialysis time (month) (*n* = 70) Median (IQR)	36 (7.5–156)	96 (13–132)	36 (6–156)	0.565 ***	120 (36–180)	15.5 (4–132)	0.003 ***	120 (5–156)	36 (10–156)	0.599 ***
Type of donation										
Living *n* (%)	57 (60.6%)	11 (45.8%)	46 (65.7%)	0.085 **	16 (45.7%)	41 (69.5%)	0.023 **	13 (46.4%)	44 (66.7%)	0.066 **
Cadaveric *n* (%)	37 (39.4%)	13 (54.2%)	24 (34.3%)		19 (54.3%)	18 (30.5%)		15 (53.6%)	22 (33.3%)	
Cold ischemia (min) Median (IQR)	130 (110–840)	430 (115–855)	110 (130–810)	0.572 ***	660 (120–930)	130 (110–690)	0.03 ***	645 (120–915)	125 (110–720)	0.036 ***

*: *p* value by Student’s test **: *p* value by chi-square test ***: *p* value by Mann–Whitney U test ****: *p* value by Fisher’s Exact test DM: Diabetes mellitus, HT: Hypertension, SD: Standard deviation, IQR: Interquartile range, BMI: Body mass index, SMI: Skeletal muscle index, IMAC: Intramuscular adipose tissue content, VSR: Visceral-to-subcutaneous adipose tissue ratio, e-GFR: Estimated glomerular filtration rate.

**Table 2:** Graft outcomes and mortality rates according to SMI, IMAC, and VSR classifications.

		SMI			IMAC			VSR		
	All (*n* = 94)	Low (*n* = 24)	Normal (*n* = 70)	*p*	High (*n* = 35)	Normal (*n* = 59)	*p*	High (*n* = 28)	Normal (*n* = 66)	*p*
Short-Term Graft Outcomes										
Acute rejection *n* (%)	26 (27.7%)	6 (25.0%)	20 (28.6%)	0.736 *	11 (31.4%)	15 (25.4%)	0.529 *	6 (21.4%)	20 (30.3%)	0.379 *
Early graft failure *n* (%)	6 (6.4%)	2 (8.3%)	4 (5.7%)	0.651 *	5 (14.3%)	1 (1.7%)	0.026 **	1 (3.6%)	5 (7.6%)	0.665 **
Delayed graft function *n* (%)	10 (10.6%)	4 (16.7%)	6 (8.6%)	0.271 **	8 (22.9%)	2 (3.4%)	0.005 **	5 (17.9%)	5 (7.6%)	0.157 **
Long-term Graft Outcomes										
Graft function decline (*n* = 86) *n* (%)	17 (19.8%)	5 (22.7%)	12 (18.8%)	0.759 **	5 (17.9%)	12 (20.7%)	1 **	8 (30.8%)	9 (15.0%)	0.092 *
Death with a functioning graft *n* (%)	5 (5.3%)	0 (0.0%)	5 (7.1%)	0.324 **	2 (5.7%)	3 (5.1%)	1 **	2 (7.1%)	3 (4.5%)	0.632 **
Death-censored graft failure *n* (%)	10 (10.6%)	3 (12.5%)	7 (10.0%)	0.712 **	7 (20.0%)	3 (5.1%)	0.036 **	3 (10.7%)	7 (10.6%)	1 **
Overall graft failure *n* (%)	15 (16.0%)	3 (12.5%)	12 (17.1%)	0.753 **	9 (25.7%)	6 (10.2%)	0.047 **	5 (17.9%)	10 (15.2%)	0.763 **
Mortality (1 year) (*n* = 78) *n* (%)	4 (5.1%)	1 (5.3%)	3 (5.1%)	1 **	4 (14.8%)	0 (0.0%)	0.012 **	1 (5.3%)	3 (5.1%)	1 **
Mortality (Overall) *n* (%)	10 (10.6%)	2 (8.3%)	8 (11.4%)	1 **	6 (17.1%)	4 (6.8%)	0.166 **	4 (14.3%)	6 (9.1%)	0.478 **

*: *p* value by Pearson chi-square test **: *p* value by Fisher’s exact test, SMI: Skeletal muscle index, IMAC: Intramuscular adipose tissue content, VSR: Visceral-to-subcutaneous adipose tissue ratio.

**Table 3:** The univariate and multivariate logistic regression results of body parameters and graft outcomes.

**Early Graft Failure**				
	**Univariate OR (95% CI)**	***p* Value**	**Adjusted OR (95% CI)**	***p* Value**
Low-SMI vs. Normal SMI	1.5 (0.3–8.6)	0.652	-	
High-IMAC vs. Normal IMAC	9.7 (1.1–86.5)	0.042	1.23 (0.1–22.9) *	0.888
High VSR vs. Normal VSR	2.2 (0.2–19.9)	0.478	-	
**Delayed graft function**				
Low-SMI vs. Normal SMI	2.1 (0.5–8.3)	0.275	-	
High-IMAC vs. Normal IMAC	8.4 (1.7–42.5)	0.010	5.5 (0.9–34.2) **	0.065
High VSR vs. Normal VSR	2.7 (0.7–10.1)	0.150	2.6 (0.5–13.3) **	0.256
**Death-censored graft failure**				
Low-SMI vs. Normal SMI	1.3 (0.3–5.4)	0.732	-	
High-IMAC vs. Normal IMAC	4.7 (1.1–19.4)	0.034	2.8 (0.5–17.2) ***	0.271
High VSR vs. Normal VSR	1.1 (0.2–4.2)	0.988	-	
**Overall graft failure**				
Low-SMI vs. Normal SMI	0.7 (0.2–2.7)	0.594		
High-IMAC vs. Normal IMAC	3.1 (0.9–9.5)	0.054	2.2 (0.5–9.3) ****	0.317
High VSR vs. Normal VSR	1.2 (0.4–4.0)	0.743		
**Mortality (1 year) (n = 78)**				
Low-SMI vs. Normal SMI	0.9 (0.1–9.8)	0.980	-	
High-IMAC vs. Normal IMAC	280.9 (0.1–486.2)	0.997	-	
High VSR vs. Normal VSR	0.8 (0.8–7.8)	0.831	-	

*: Adjusted with recipient age, donor age, cold ischemia time, dialysis duration **: Adjusted with dialysis duration, cold ischemia time ***: Adjusted with recipient age, donor age, cold ischemia time, dialysis duration, donation type ****: Adjusted with recipient age, donor age, BMI, cold ischemia time, dialysis duration, operation side, donation type, SMI: Skeletal muscle index, IMAC: Intramuscular adipose tissue content, VSR: Visceral-to-subcutaneous adipose tissue ratio.

## Data Availability

Data supporting the findings of this study can be obtained, but there are restrictions on the availability of these data. These data were used under license for the current study and are therefore not publicly available. However, data can be obtained from the corresponding author upon reasonable request.

## Abbreviations

RT	Renal transplantation
ESRF	End-stage renal failure
CT	Computed tomography
VSR	Visceral-to-subcutaneous adipose tissue ratio
SMI	Skeletal muscle index
IMAC	Intramuscular adipose tissue content
SMA	Skeletal muscle area
KTRs	Kidney transplant recipients
BMI	Body mass index
HU	Hounsfield unit
DM	Diabetes mellitus
HT	Hypertension
CVD	Cardiovascular diseases
SD	Standard deviation
IQR	Interquartile range
e-GFR	Estimated glomerular filtration rate
MS	Metabolic syndrome
VAT	Visceral adipose tissue
